# Family involvement and patient-experienced improvement and satisfaction with care: a nationwide cross-sectional study in Danish psychiatric hospitals

**DOI:** 10.1186/s12888-021-03179-1

**Published:** 2021-04-13

**Authors:** Marie Louise Svendsen, Trine Ellegaard, Karoline Agerbo Jeppesen, Erik Riiskjær, Berit Kjærside Nielsen

**Affiliations:** 1 DEFACTUM, Central Denmark Region, Olof Palmes Allé 15, 8200 Aarhus, Denmark; 2 Aarhus University Hospital, Psychiatry, Psychosis Research Unit, Palle Juul-Jensens Boulevard 175, 8200 Aarhus, Denmark; 3 The Research Centre for Patient Involvement, Aarhus University & the Central Denmark Region, Bartholins Allé 2, 8000 Aarhus, Denmark; 4 Aarhus University, Faculty of Health, Vennelyst Boulevard 4, 8000 Aarhus, Denmark

**Keywords:** Psychiatry, Caregivers, Patient reported outcome measures, Patient reported experience measures, Patient satisfaction

## Abstract

**Background:**

Randomised controlled trials suggest that family therapy has a positive effect on the course of depression, schizophrenia and anorexia nervosa. However, it is largely unknown whether a positive link also exists between caregiver involvement and patient outcome in everyday psychiatric hospital care, using information reported directly from patients, i.e. patient-reported experience measures (PREM), and their caregivers. The objective of this study is to examine whether caregiver-reported involvement is associated with PREM regarding patient improvement and overall satisfaction with care.

**Methods:**

Using data from the National Survey of Psychiatric Patient Experiences 2018, we conducted a nationwide cross-sectional study in Danish psychiatric hospitals including patients and their caregivers who had been in contact with the hospital (*n* = 940 patients, *n* = 1008 caregivers). A unique patient identifier on the two distinct questionnaires for the patient and their caregiver enabled unambiguous linkage of data. In relation to PREM, five aspects of caregiver involvement were analysed using logistic regression with adjustment for patient age, sex and diagnosis.

**Results:**

We consistently find that high caregiver-reported involvement is statistically significantly associated with high patient-reported improvement and overall satisfaction with care with odds ratios (OR) ranging from 1.69 (95% confidence interval (CI) 0.95–2.99) to 4.09 (95% CI 2.48–6.76). This applies to the following aspects of caregiver-reported involvement: support for the patient-caregiver relationship, caregiver information, consideration for caregiver experiences and the involvement of caregivers in decision making. No statistically significant association is observed regarding whether caregivers talk to the staff about their expectations for the hospital contact.

**Conclusion:**

This nationwide study implies that caregiver involvement focusing on the patient-caregiver relationship is positively associated with patient improvement and overall satisfaction with care in everyday psychiatric hospital care.

**Supplementary Information:**

The online version contains supplementary material available at 10.1186/s12888-021-03179-1.

## Background

Today, most mental health treatment guidelines aim to enhance recovery-orientated psychiatric care by encouraging the systematic involvement of patients and their family or peer relatives (from here on caregivers) in treatment planning and decision making [1]. Studies show that people with mental disorders have better treatment outcomes when a caregiver is involved in their care. Caregivers may have a positive impact on a patient’s health by providing moral and emotional support, practical support and motivation to recover and by facilitating healthy behaviour through, e.g. compliance with medical treatment [2–4]. Moreover, caregivers may also be perceived as a source of information regarding the patient’s situation [5]. As a consequence, there is a growing scientific and clinical focus on caregiver involvement as a resource in the recovery from mental disorders [3, 6].

In a historical perspective, caregiver involvement and the role of caregiver support in the recovery from mental disorders have gained little attention. An influential study from 1972 shows that patients with schizophrenia from families with high expressed emotions (defined as having high levels of critical comments, hostility and emotional over-involvement) have relapses more frequently than patients from families with fewer expressed emotions [7]. Furthermore, studies suggest that families may impede recovery for persons with mental disorders by acting as a stressor and displaying stigma and lack of understanding [3]. Several interventions targeting the family are now available, and randomised controlled trials suggest that family therapy compared to standard treatment has a positive effect on the course of depression, schizophrenia and anorexia nervosa [2, 6, 8]. Other studies further indicate that caregivers may mediate and moderate the impact of the prognostic factors of mental disorder, including the effect of clinical interventions [9, 10].

The World Psychiatric Association recommends that acute or rehabilitation situations in psychiatric care are managed in collaboration between mental health professionals, patients and caregivers to provide the best psychiatric care. The involvement of the patients and their caregivers should undergo quality assurance by monitoring, evaluating and disseminating the results [11]. Furthermore, international guidelines from the American Psychiatry Association and the National Institute for Health and Care Excellence specifically state that the involvement of caregivers is good clinical practice in psychiatric care. Clinical guidelines may be implemented in everyday clinical practice through large-scale quality improvement initiatives. However, only limited research has focused on the involvement of caregivers in psychiatric care in the context of quality improvement programmes. Two nationwide population-based Danish studies, using data from a nationwide quality improvement programme for in-hospital care of patients with schizophrenia, show that the fulfilment of quality indicators expressing the proportion of staff-reported contact with caregivers increased from 47% in 2004 to 67% in 2011 and that staff-reported contact with caregivers is associated with reduced risk of criminal behaviour after discharge [12, 13].

However, the monitoring and evaluation of the quality of care have primarily focused on clinical performance based on reports from health care professionals although that patients’ experiences, including patients’ satisfaction with care, have been recognised as one of the three pillars of quality in health care along with clinical effectiveness and patient safety [14, 15]. Patient-reported experience measures (PREMs) assess patients’ experiences with health care and may therefore hold valuable information regarding patient-centred care. Contrary to patient-reported outcome measures, PREMs have received less attention in research, particularly regarding the association between PREMs and other quality measures [16, 17]. The validity of PREMs has primarily been justified by its intrinsic value because it reflects the authoritative and unique insight into the patients’ experiences of all aspects of care. Nevertheless, emerging evidence suggests that higher levels of positive patient experiences are associated with more favourable health outcomes [14]. However, most research covers non-psychiatric diseases, and research is scarce regarding the value of using PREMs in psychiatric care [14, 17].

To the best of our knowledge, no prior studies have assessed whether the fulfilment of quality measures concerning caregivers’ perception of involvement is associated with patient-experienced improvement and satisfaction with psychiatric care [17]. Such knowledge is essential to verify whether a positive link between caregiver involvement and patient outcome also exists in everyday psychiatric hospital care from a patient-centred perspective.

## Methods

### Aims of the study

We examined whether caregivers’ perception of five aspects of caregiver involvement during inpatient and outpatient psychiatric hospital care is associated with two patient-reported experience measures regarding improvement and overall satisfaction with care.

### Design and setting

We conducted a nationwide, cross-sectional study applying data from the National Survey of Psychiatric Patient Experiences 2018, covering all Danish public psychiatric hospitals. We included patients ≥18-years-old receiving inpatient (week 36–45, 2018) and outpatient (week 36–38, 2018) care and their appointed caregivers. Patients receiving inpatient forensic psychiatric care were not included. The Danish health care system is a public, mainly tax-funded healthcare system with free access to hospital care for all Danish residents [18]. Public hospitals (somatic and psychiatric) and local community mental health centres are owned and operated by the five regions in Denmark [18]. If hospitalisation is required, patients with a psychiatric disorder have free access to psychiatric hospital care.

### The National Survey of psychiatric patient experiences

The National Survey of Psychiatric Patient Experiences was established in 2005 with the objective of monitoring, reporting and improving the quality of care from the perspective of patients and their caregivers in all Danish public psychiatric hospitals. The survey has been completed annually since 2012 among patients and parents within child and adolescent psychiatry, and every third year among caregivers for adult psychiatric patients. The National Survey of Psychiatric Patient Experiences includes person-level data on PREM as well as data reported by caregivers regarding inpatient and outpatient psychiatric hospital care. The survey also includes sociodemographic and clinical data. The National Survey of Psychiatric Patient Experiences is administered by a steering group including patients and caregivers, and representatives from the Ministry of Health, the Danish Health Data Authority and the Danish Regions. Regional coordinators and local facilitators in each hospital unit ensure that the health care professionals receive written standardised guidelines for the inclusion of patients and caregivers, and the data collection. Each year, data collection is carried out during a fixed period running from week 36 to week 38 for adult psychiatric outpatients, and from week 36 to week 45 for adult psychiatric inpatients. All Danish public psychiatric hospital departments are invited to participate in the survey if they treat more than 10 patients during the inclusion period. The mental health professionals personally hand out paper questionnaires to the patients. Caregivers, appointed by the patient, receive a paper questionnaire by post. All invited patients and caregivers receive a stamped envelope to return the completed questionnaires, and electronic completion of the questionnaire is also possible. The standardised guidelines for data collection specify that only on specific request from the patients are health care professionals permitted to help the patients understand the questions. However, they should not influence or directly observe the patients’ responses. All items in the questionnaires for patients and caregivers are continuously validated (face validity) by semi-structured interviews with hospitalised patients and caregivers. The results from the National Survey of Psychiatric Patient Experiences 2018 were published in March 2019 [19].

### Participants

Patients are included in the National Survey of Psychiatric Patient Experiences if they have at least two outpatient contacts preceding an outpatient contact during week 36–38 (outpatient care), and if hospital discharge is planned during week 36–45 (inpatient care). These criteria imply that patients with transitory hospital contact are excluded and that patient and their caregivers are capable of responding to questions concerning the course of hospital care. Furthermore, based on a clinical judgement by the mental health professionals caring for the patients, patients are excluded if they are unable to participate in the survey because of (1) severe psychosis, (2) severe dementia, (3) moderate to severe mental retardation, (4) being moribund, or (5) being acutely transferred to a somatic hospital. Patients who give consent to participate may appoint one or two caregivers for inclusion in the survey. A unique patient identifier on each questionnaire for the patient and their caregiver enables the unambiguous linkage of the data between them.

In 2018, a total of 447 hospital departments participated in the National Survey of Psychiatric Patient Experiences representing all Danish psychiatric hospitals. The response rate was 60% (*n* = 6229) among adult outpatients, 38% (*n* = 1460) among their invited caregivers, 69% (*n* = 2010) among adult inpatients and 36% (*n* = 216) among their invited caregivers [19]. Because 506 outpatients and 97 inpatients had two caregivers included in the survey, the total number of patient records are 10803 for outpatient care and 3029 for inpatient care (cf. Supplementary material, Additional file 1). In the National Survey of Psychiatric Patient Experiences, the caregivers are only directed to respond to questions about involvement if they have been in contact with the mental health professionals by telephone or in person, i.e. the items applied in this study. Therefore, this study only includes caregivers who specifically stated that they had been in contact with the mental health professionals. Furthermore, this study only includes patients and caregivers who had completed a questionnaire, leaving a total of 791 patients and 846 caregivers in outpatient psychiatric care representing 147 hospital units and 149 patients and 162 caregivers in inpatient psychiatric care representing 69 hospital units (cf. Supplementary material, Additional file 1).

### Caregiver involvement, PREM, and patient characteristics

Table 1 shows the items and response scales applied in the National Survey of Psychiatric Patient Experiences to assess caregivers’ perception of involvement and patient-reported improvement and overall satisfaction with care. The five items for caregiver involvement were reported by the caregivers on a dedicated caregiver questionnaire, and the two items for PREMs regarding improvement and overall satisfaction with care were reported by the patients on a dedicated patient questionnaire. Baseline characteristics regarding patient age, patient sex, eight main diagnosis categories, and caregiver relationship were registered by the mental health professionals, cf. Table 2.

**Table 1:** Items for caregivers-reported involvement and patient-reported experience measures

Survey	Item text
Caregiver	
	Item 1: Is it your impression that the staff support the patient in having contact with their caregivers?^a^
	Item 2: Do you receive the information about the patient’s disease and treatment that you need?^a^
	Item 3: Do you talk to the staff about your expectations for the hospital contact?^a^
	Item 4: Do the staff ask about your own experiences with the patient’s disease and/or condition?^a^
	Item 5: Are you sufficiently involved in decisions about the patient’s examination and/or treatment?^b^
Patients	
	Item 1: Did you get better due to the hospitalisation (inpatients)/treatment in the hospital department (outpatients)? ^a^
	Item 2: All things considered, are you satisfied with the hospitalisation (inpatients) /hospital contact (outpatients)?^a^

^a^ Response scale: Five-point Likert scale (‘very high degree’, ‘high degree’, ‘some degree’, ‘low degree’, ‘not at all’)

^b^ Response scale: ‘Yes’, ‘No’

**Table 2:** Descriptive patient characteristics (1008 patient records, 940 patients)

Characteristics	Patient-reported improvement^**a**^				Patient-reported satisfaction^**b**^			
	Outpatient care		Inpatient care		Outpatient care		Inpatient care	
	High, *n* = 514	Low, *n* = 286	High, *n* = 106	Low, *n* = 47	High, *n* = 712	Low, *n* = 112	High, *n* = 107	Low, *n* = 45
**Age, m (SD)**	37.2 (17.1)	38.6 (17.8)	48.8 (19.4)	40.6 (17.7)	37.3 (17.1)	37.8 (18.1)	48.5 (20.0)	42.3 (16.8)
Age missing, n (%)	7 (1.4)	3 (1.0)	1 (0.9)	0 (0)	8 (1.1)	1 (0.9)	1 (0.9)	1 (2.2)
**Sex, n (%)**								
Male	201 (39.1)	87 (30.4)	46 (43.4)	20 (42.6)	262 (36.8)	37 (33.0)	45 (42.1)	19 (42.2)
Female	306 (59.5)	194 (67.8)	54 (50.9)	26 (55.3)	438 (61.5)	74 (66.1)	56 (52.3)	25 (55.6)
Missing	7 (1.4)	5 (1.8)	6 (5.7)	1 (2.1)	12 (1.7)	1 (0.9)	6 (5.6)	1 (2.2)
**Diagnosis, n (%)**								
Schizophrenia and psychosis	238 (46.3)	85 (29.7)	23 (21.7)	11 (23.4)	297 (41.7)	34 (30.4)	22 (20.6)	10 (22.2)
Affective disorder	130 (25.3)	68 (23.8)	57 (53.8)	14 (29.8)	178 (25.0)	23 (20.5)	56 (52.3)	17 (37.8)
Other diagnosis^c^	138 (26.9)	128 (44.8)	24 (22.6)	21 (44.7)	225 (31.6)	54 (48.2)	26 (24.3)	17 (37.8)
Missing	8 (1.6)	5 (1.8)	2 (1.9)	1 (2.1)	12 (1.7)	1 (0.9)	3 (2.8)	1 (2.2)
**Relationship, n (%)**								
Partner	161 (31.3)	100 (35.0)	42 (39.6)	13 (27.7)	233 (32.7)	32 (28.6)	37 (34.6)	16 (35.6)
Parent	269 (52.3)	138 (48.3)	33 (31.1)	21 (44.7)	364 (51.1)	58 (51.8)	36 (33.6)	17 (37.8)
Son/daughter	41 (8.0)	29 (10.1)	23 (21.7)	8 (17.0)	55 (7.7)	14 (12.5)	26 (24.3)	7 (15.6)
Sibling	18 (3.5)	9 (3.2)	2 (1.9)	2 (4.3)	24 (3.4)	5 (4.5)	2 (1.9)	2 (4.4)
Other relation	25 (4.9)	9 (3.2)	6 (5.7)	2 (4.3)	35 (4.9)	3 (2.7)	6 (5.6)	2 (4.4)
Missing	0 (0)	1 (0.4)	0 (0)	1 (2.1)	1 (0.1)	0 (0)	0 (0)	1 (2.2)

^a^ Patient-reported improvement, missing data: *n* = 46 (5.4%, outpatient), *n* = 9 (5.6%, inpatient)

^b^ Patient-reported satisfaction, missing data: *n* = 22 (2.6%, outpatient), *n* = 10 (6.1%, inpatient)

^c^ Includes: organic mental disorders, mental and behavioural disorders due to psychoactive substance use, neurotic, stress-related and somatoform disorders, behavioural syndromes associated with physiological disturbances and physical factors, disorders of adult personality and behaviour, and other disorder/symptom (e.g. unclear diagnosis)

### Statistical analysis

The 5-point response scale for caregiver involvement and patient-reported improvement and overall satisfaction with care was categorised into high (‘very high degree’, ‘high degree’) and low/none (‘some degree’, ‘low degree’, ‘not at all’). Age was applied in the analysis as a continuous variable, and the remaining baseline characteristics were categorised, as stated in Table 2. The association between the specific items for caregiver involvement and patient-reported improvement as well as satisfaction with care were analysed using logistic regression adjusting for patient age, sex, and diagnosis. Patients were included twice if they had two caregivers included in the study. Therefore, the unadjusted and the adjusted analyses allowed for patient-level clustering using cluster-robust standard errors. Only caregivers defining the specific aspect of caregiver involvement relevant to them were included in the analyses and, therefore, the specific number of observations in the logistic regression analyses varied (cf. Supplementary material, Additional file 2). The analyses were performed as complete-case analyses with two-tailed testing using a significance level of 0.05.

A sensitivity analysis was performed to evaluate whether regional differences in hospital ownership and operation could possibly influence the results by repeating the primary adjusted logistic regression analyses, including regional affiliation as an extra covariate. Furthermore, descriptive statistics were performed comparing patients with no appointed relatives, appointed non-responding relatives and appointed responding relatives with regard to baseline characteristics and patient-reported improvement and overall satisfaction with care. Data were analysed with Stata 16 (College Station, TX: StataCorp LLC).

## Results

### Descriptive statistics

The study includes 940 patients and 1008 caregivers because 55 outpatients and 13 inpatients have two caregivers included in the study. As shown in Table 2, the caregiver relationship is either a partner or a parent for approximately 80% of the patients receiving outpatient care and for 70% of the patients receiving inpatient care. Patients with other psychiatric diagnoses than schizophrenia, psychotic disorders or affective disorders generally tend to report less improvement and satisfaction with care than patients with these diagnoses.

A high proportion of caregivers report low/none involvement in patient care ranging from 20.4% (outpatients) for the item ‘Is it your impression that the staff supports the patient in having contact with their caregivers?’ to 72.2% (inpatients) for the item ‘Do you talk to the staff about your expectations for the hospital contact?’ (cf. Supplementary material, Additional file 2). Most caregivers identify the specific aspects of caregiver involvement relevant to them. Still, up to 26% of the caregivers state that involvement in decision making is not relevant to them (cf. Supplementary material, Additional file 2).

### Patient-reported improvement

As shown in Table 3, no statistically significant association is observed between whether caregivers talk to the mental health professionals about their expectations for hospital contact and patient-reported improvement. The point estimates for the remaining four aspects of caregiver involvement point towards a positive association between a high degree of caregiver involvement and high patient-reported improvement compared with less or no caregiver involvement. However, only six out of the eight point estimates reach statistical significance. Among patients in outpatient care, a strong association is observed between a high degree of caregiver involvement and high patient-reported improvement when the mental health professionals support the patient in having contact with their caregivers (adjusted odds ratio (OR) 2.19, 95% Confidence Interval (CI) 1.49–3.22). Among inpatients, a strong association between a high degree of caregiver involvement and high patient-reported improvement is observed for the aspect of caregiver involvement assessing whether the caregivers are sufficiently involved in decision making (adjusted OR 3.39, 95% CI 1.35–8.47).

**Table 3:** Association between caregiver involvement and patient-reported improvement

	Outpatient care: high patient-reported improvement			Inpatient care: high patient-reported improvement		
Caregiver involvement^a^	n (%)^b^	Crude OR (95% CI)	Adjusted OR (95% CI)^c^	n (%)^b^	Crude OR (95% CI)	Adjusted OR (95% CI)^c^
Staff support patient in having contact with caregivers						
- High	360 (69.1)	2.10 (1.46–3.04)	2.19 (1.49–3.22)	65 (76.5)	2.34 (1.02–5.36)	2.55 (1.01–6.42)
- Low/none	84 (51.5)	Reference group	Reference group	25 (58.1)	Reference group	Reference group
Sufficient information about disease and treatment						
- High	233 (71.9)	1.88 (1.36–2.58)	1.72 (1.23–2.42)	36 (80.0)	2.25 (0.99–5.13)	2.31 (0.90–5.94)
- Low/none	236 (57.7)	Reference group	Reference group	64 (64.0)	Reference group	Reference group
Talk to staff about expectations						
- High	85 (71.4)	1.57 (1.00–2.47)	1.37 (0.84–2.23)	17 (68.0)	1.03 (0.40–2.67)	1.23 (0.46–3.28)
- Low/none	331 (61.4)	Reference group	Reference group	74 (67.3)	Reference group	Reference group
Staff ask about your experiences						
- High	187 (73.1)	1.97 (1.40–2.76)	1.78 (1.24–2.54)	31 (81.6)	2.50 (0.92–6.83)	2.67 (0.92–7.74)
- Low/none	274 (57.9)	Reference group	Reference group	69 (63.9)	Reference group	Reference group
Sufficiently involved in decision making						
- Yes	185 (70.1)	1.94 (1.33–2.82)	1.90 (1.28–2.83)	43 (81.1)	4.01 (1.69–9.51)	3.39 (1.35–8.47)
- No	140 (54.7)	Reference group	Reference group	30 (51.7)	Reference group	Reference group

^a^ The 5-point response scale for caregiver involvement categorised into high (‘very high degree’, ‘high degree’) and low/none (‘some degree’, ‘low degree’, ‘not at all’)

^b^ The number and percentage of high patient-reported improvement in a specific group of caregiver involvement. The inverse percentage, that adds up to 100%, represents the patient records with low/none patient-reported improvement in the specific group of caregiver involvement (not shown in the table)

^c^ Adjusted for patient age, sex and diagnosis

### Patient-reported satisfaction

In general, similar tendencies are observed between caregiver involvement and overall patient-reported satisfaction with care, cf. Table 4. No statistically significant association is observed between whether caregivers talk to the professionals about their expectations and patient-reported satisfaction. The ORs for the remaining four aspects of caregiver involvement range between 1.69 (95% CI 0.95–2.99) and 4.09 (95% CI 2.48–6.76), cf. Table 4.

**Table 4:** Association between caregiver involvement and patient-reported satisfaction with care

	Outpatient care: high patient-reported satisfaction			Inpatient care: high patient-reported satisfaction		
Caregiver involvement^a^	n (%)^b^	Crude OR (95% CI)	Adjusted OR (95% CI)^c^	n (%)^b^	Crude OR (95% CI)	Adjusted OR (95% CI)^c^
Staff support patient in having contact with caregivers						
- High	490 (91.6)	3.75 (2.32–6.06)	4.09 (2.48–6.76)	64 (76.1)	2.53 (1.10–5.81)	3.22 (1.25–8.29)
- Low/none	125 (74.4)	Reference group	Reference group	24 (55.8)	Reference group	Reference group
Sufficient information about disease and treatment						
- High	303 (92.7)	2.85 (1.73–4.70)	2.75 (1.64–4.60)	38 (82.6)	2.76 (1.18–6.45)	2.63 (1.04–6.64)
- Low/none	350 (81.5)	Reference group	Reference group	62 (63.3)	Reference group	Reference group
Talk to staff about expectations						
- High	112 (90.3)	1.70 (0.86–3.38)	1.76 (0.84–3.68)	18 (69.2)	1.06 (0.41–2.73)	0.98 (0.38–2.52)
- Low/none	471 (84.6)	Reference group	Reference group	74 (67.9)	Reference group	Reference group
Staff ask about your experiences						
- High	240 (92.7)	2.59 (1.50–4.48)	2.47 (1.41–4.32)	33 (84.6)	3.20 (1.09–9.36)	3.40 (1.14–10.18)
- Low/none	409 (83.0)	Reference group	Reference group	67 (63.2)	Reference group	Reference group
Sufficiently involved in decision making						
- Yes	244 (89.1)	1.80 (1.06–3.06)	1.69 (0.95–2.99)	45 (81.8)	4.03 (1.70–9.56)	3.31 (1.37–7.99)
- No	212 (81.9)	Reference group	Reference group	29 (52.7)	Reference group	Reference group

^a^ The 5-point response scale for caregiver involvement categorised into high (‘very high degree’, ‘high degree’) and low/none (‘some degree’, ‘low degree’, ‘not at all’)

^b^ The number and percentage of high patient-reported improvement in a specific group of caregiver involvement. The inverse percentage, that adds up to 100%, represents the patient records with low/none patient-reported improvement in the specific group of caregiver involvement (not shown in the table)

^c^ Adjusted for patient age, sex and diagnosis

### Sensitivity analysis

The sensitivity analysis shows that adjustment for regional affiliation, thereby taking into account potential differences in hospital ownership and operation, had only a minor influence on the point estimates but, as expected, widened the CIs (cf. Supplementary material, Additional file 3). Furthermore, descriptive characteristics for patients with no appointed caregivers, non-responding caregivers and responding caregivers reveal some differences according to caregiver participation. There is a tendency that patients with appointed caregivers (responding and non-responding) generally report similar or higher improvement and satisfaction with care than patients with no appointed caregivers. There are also some differences in patient diagnoses according to caregiver participation (cf. Supplementary material, Additional files 4 and 5).

## Discussion

Caregivers are increasingly acknowledged and identified as necessary collaborators as a result of their contributions to health and the provision of health care [20]. The findings in this study also imply that for outpatients and inpatients, the aspect of caregiver involvement focusing on supporting the contact between the patients and their caregivers is important. Increasing evidence highlights the benefit of supporting people to manage their own health as effectively as possible and draws attention to the social context of self-management support [21]. The purpose of self-management support is to aid and encourage patients to make daily decisions that improve health-related behaviours, enabling them to recognise and develop their own strengths and their ability to live independently [20]. An important aspect of this aid is that caregivers can help facilitate and support the patients and the professionals. However, in clinical mental health care, it can be challenging to involve caregivers if patients decline to consent to their involvement. Barriers for the involvement of caregivers related to persons with severe mental disorders may include not getting along with the caregivers, unavailability of caregivers or concerns about burdening the caregivers [22].

A strong association is found, particularly among inpatients, when the caregivers report being involved in decisions concerning the patient’s treatment and care, indicating that caregivers may play a vital role. However, research shows that collaborative decision making is not often experienced by caregivers and that professionals can have a negative attitude towards involving caregivers in care planning [1]. Furthermore, the professionals may lack a judicial understanding of confidentiality, resulting in the exclusion of caregivers from the care planning process [1]. This may cause a negative dynamic and a power struggle between caregivers and health care professionals and may constitute an assumable risk of impeding the patients’ recovery process [1]. Furthermore, organisational culture and paradigms can work to limit the involvement of caregivers, and health care professionals in clinical practice have a key function in involving patients and their caregivers in treatment [23]. Mental health professionals need the right organizational infrastructure and tools to make caregiver involvement standard every day practice [24].

No association is observed in this study for the aspect of caregiver involvement focusing on the caregivers’ expectations for the hospital contact. This aspect of caregiver involvement differs from the remaining four aspects in several ways, which may explain the inconsistent results. First, asking about caregiver expectations may be perceived as a more far-reaching and comprehensive aspect of caregiver involvement than the remaining four aspects. Second, staff questions about caregivers’ expectations for an upcoming contact are not aimed directly at the caregiver-patient relationship, unlike the remaining four aspects of caregiver involvement. However, empirical studies indicate that caregivers experience a gap between their expectations and the perceptions of actual support received from professionals. Therefore, caregivers may still need support and the opportunity to express their expectations [25]. Research indicates that the caregivers can be frustrated by the absence of information [26]. However, being close to a person with mental disorders can induce physical, emotional and financial strain [27]. Therefore, there can be a discrepancy between the needs of patients and the needs of caregivers.

A main limitation of our study is the observational design. Causality between caregiver involvement and patient outcome has largely been established by previous research, and caution is particularly needed concerning the magnitude of associations [2, 6, 8]. We cannot exclude the possibility that our results may be influenced by residual confounding due to the use of crude variables (e.g. using three main diagnosis categories) or unaccounted confounding from, e.g. socioeconomic status [28]. However, adjustment for patient age, sex and main diagnosis had no substantial influence on the unadjusted point estimates indicating that confounding from these factors has only minor influence on the results. Potential confounding would most likely affect all investigated associations, and our study shows point estimates that vary in strength and statistical significance. Therefore, we believe that confounding may influence, but not fully explain the observed associations.

Another important limitation of our study is that the data collection among outpatients and their caregivers is completed during a specific inclusion period irrespective of the course of the patients’ disorder. Inpatients are only included if their discharge is planned, and this makes the clinical stage of their disorder more homogeneous. The concordant results between outpatients and inpatients may yet indicate that the stage of the specific disorders has no major influence on the observed associations. Furthermore, response errors and misclassification are a concern because data were registered by a large number of patients and caregivers. It is likely that such misclassification is non-differential and would bias the results towards the null, thereby decreasing the strength of associations [29].

An important strength of this study is the application of nationwide person-level data reported directly from the patients and caregivers representing all Danish psychiatric hospitals, thereby optimising the generalisability of the results to everyday clinical care. Furthermore, the ability to unambiguously link the data from one patient and their caregiver, and the separate responding from patients and caregivers, reduce the risk of information bias. However, it is natural in many family relationships that caregivers are aware of the patients’ improvement process and their satisfaction with their care.

Approximately 9% of outpatients and 4% of inpatients in the National Survey of Psychiatric Patient Experiences 2018 had responding caregivers, and the results of this study only apply to these patient-caregiver relationships [19]. It is plausible that caregivers who are less involved in patient care are less likely to respond to the survey and be included in this study. Therefore, it is unknown whether the demonstrated positive associations are representative of the nationwide level, and whether the demonstrated positive associations would apply if interventions were implemented to increase caregiver involvement in the care of patients in psychiatric hospitals. Therefore, although our data directly reflect the patient and caregiver experiences of everyday psychiatric hospital care, the results need to be verified in other populations and settings to confirm their general applicability.

## Conclusions

Supported by previous research, this study calls for the systematic involvement of caregivers in psychiatric hospital care. It emphasises the need for future research focusing on identifying and implementing core aspects of caregiver involvement and on health equality for patients with no existing, or no involved, caregivers.

## Supplementary Information

**Additional file 1.** Flowchart. Detailed flowchart for inpatients and outpatients.

**Additional file 2.** Descriptive data about caregiver involvement. Detailed data on caregiver responses to questions regarding involvement.

**Additional file 3.** Results of a sensitivity analysis evaluating potential regional differences. Results of a sensitivity analysis evaluating whether regional differences in hospital ownership and operation could possibly influence the results by repeating the primary adjusted logistic regression analyses including regional affiliation as an extra covariate.

**Additional file 4.** Results of a sensitivity analysis according to non-appointed, non-responding and responding caregivers (outpatients). Results of a sensitivity analysis comparing descriptive statistics between patients with no appointed caregivers, appointed non-responding caregivers and appointed responding caregivers with regard to baseline characteristics and patient-reported improvement and overall satisfaction with care. This file only includes patients receiving outpatient care.

**Additional file 5.** Results of a sensitivity analysis according to non-appointed, non-responding and responding caregivers (inpatients). Results of a sensitivity analysis comparing descriptive statistics between patients with no appointed caregivers, appointed non-responding caregivers and appointed responding caregivers with regard to baseline characteristics and patient-reported improvement and overall satisfaction with care. This file only includes patients receiving inpatient care.

## Data Availability

The data that support the findings of this study are available from the National Survey of Psychiatric Patient Experiences. However, data sharing is not allowed because of Danish legal requirements.

## Abbreviations

PREM: Patient-reported experience measure
OR: Odds ratio
CI: Confidence interval
