# Supplementary material for: Family involvement and patient-experienced improvement and satisfaction with care: a nationwide cross-sectional study in Danish psychiatric hospitals
Source: BMC Psychiatry. 2021 Apr 13;21:190. doi: 10.1186/s12888-021-03179-1 (PMC8042926; doi:10.1186/s12888-021-03179-1)

Additional file 1. Flowchart

Figure 1. Outpatient care (adult psychiatric patients)

Patient records
n=10,803

- No invited caregivers, n=6,982
- 1 caregiver, n=2,809
- 2 caregivers, n=1,012

Unique patients
n=10,297 (=6,982+2,809+(1,012/2))

Invited caregivers, n=3,821

- Responding, n=1,460
- Non-responding, n=2,361

Non-responding patients (records)
n=4,203

Responding patient
Patient records, n=6,600
Unique patients, n=6,229

Patients with no invited caregivers (records)
n=4,018

Patients with invited caregivers
Patient records, n=2,582
Unique patients, n=2,211

Patients with non-responding caregivers (records)
n=1,484

Patients with responding caregivers
Patient records, n=1,098
Unique patients, n=1,013

Patients with caregivers having no contact with the mental health professionals (records)
n=252

Patients with responding caregivers
Patient records, n=846
Unique patients, n=791

Figure 2. Inpatient care (adult psychiatric patients)

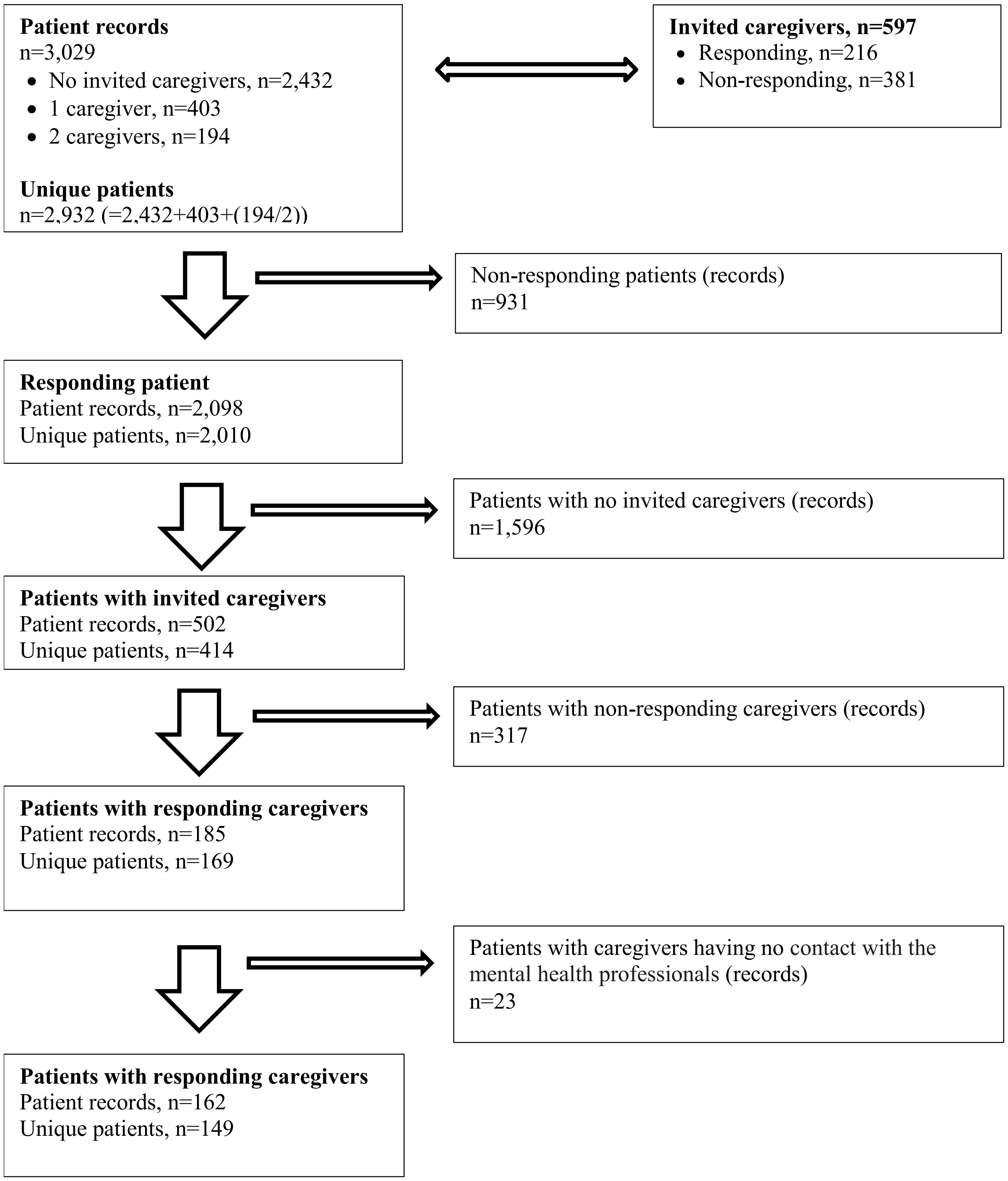

Supplement: Supplementary file 1 — Additional file 1. Flowchart. Detailed flowchart for inpatients and outpatients. [file 12888_2021_3179_MOESM1_ESM.pdf]
