# Supplementary material for: Family involvement and patient-experienced improvement and satisfaction with care: a nationwide cross-sectional study in Danish psychiatric hospitals
Source: BMC Psychiatry. 2021 Apr 13;21:190. doi: 10.1186/s12888-021-03179-1 (PMC8042926; doi:10.1186/s12888-021-03179-1)
Supplement: Supplementary file 2 — Additional file 2. Descriptive data about caregiver involvement. Detailed data on caregiver responses to questions regarding involvement. [file 12888_2021_3179_MOESM2_ESM.pdf]

Additional file 2. Descriptive data about caregiver involvement

Table. Descriptive data about caregiver involvement

Caregiver involvement	Outpatient care, n=846	Inpatient care, n=162
**Staff supports patient in having contact with caregivers, n (%)**		
Not at all	26 (3.1)	7 (4.3)
Low degree	33 (3.9)	10 (6.2)
Some degree	114 (13.5)	29 (17.9)
High degree	268 (31.7)	45 (27.8)
Very high degree	278 (32.9)	43 (26.5)
Not relevant	27 (3.2)	2 (1.2)
Unknown	100 (11.8)	26 (16.1)
**Sufficient information about disease and treatment, n (%)**		
Not at all	122 (14.4)	30 (18.5)
Low degree	113 (13.4)	25 (15.4)
Some degree	200 (23.6)	50 (30.9)
High degree	204 (24.1)	33 (20.4)
Very high degree	134 (15.8)	16 (9.9)
Not relevant	30 (3.6)	1 (0.6)
Unknown	43 (5.1)	7 (4.3)
**Talk to staff about expectations, n (%)**		
Not at all	267 (31.6)	51 (31.5)
Low degree	124 (14.7)	22 (13.6)
Some degree	177 (20.9)	44 (27.2)
High degree	90 (10.6)	20 (12.4)
Very high degree	37 (4.4)	6 (3.7)
Not relevant	83 (9.8)	8 (4.9)
Unknown	68 (8.0)	11 (6.8)
**Staff asks about your experiences, n (%)**		
Not at all	204 (24.1)	38 (23.5)
Low degree	93 (11.0)	25 (15.4)
Some degree	204 (24.1)	51 (31.5)
High degree	161 (19.0)	27 (16.7)
Very high degree	108 (12.8)	14 (8.6)
Not relevant	38 (4.5)	1 (0.6)
Unknown	38 (4.5)	6 (3.7)
**Sufficiently involved in decision making, n (%)**		
No	261 (30.9)	61 (37.7)
Yes	288 (34.0)	57 (35.2)
Not relevant	221 (26.1)	34 (21.0)
Unknown	76 (9.0)	10 (6.2)
