# Supplementary material for: Family involvement and patient-experienced improvement and satisfaction with care: a nationwide cross-sectional study in Danish psychiatric hospitals
Source: BMC Psychiatry. 2021 Apr 13;21:190. doi: 10.1186/s12888-021-03179-1 (PMC8042926; doi:10.1186/s12888-021-03179-1)
Supplement: Supplementary file 3 — Additional file 3. Results of a sensitivity analysis evaluating potential regional differences. Results of a sensitivity analysis evaluating whether regional differences in hospital ownership and operation could possibly influence the results by repeating the primary adjusted logistic regression analyses including regional affiliation as an extra covariate. [file 12888_2021_3179_MOESM3_ESM.pdf]

Additional file 3. Results of a sensitivity analysis evaluating potential regional differences

Table 1a. Descriptive characteristics of patients in Danish psychiatric hospitals (1008 patient records, 940 patients): incl. region

**Characteristics**	**Patient-reported improvement[a]**				**Patient-reported satisfaction[b]**			
	Outpatient care		Inpatient care		Outpatient care		Inpatient care	
	High, n=514	Low, n=286	High, n=106	Low, n=47	High, n= 712	Low, n=112	High, n=107	Low, n=45
**Age, m (sd)**	37.2 (17.1)	38.6 (17.8)	48.8 (19.4)	40.6 (17.7)	37.3 (17.1)	37.8 (18.1)	48.5 (20.0)	42.3 (16.8)
Age missing, n (%)	7 (1.4)	3 (1.0)	1 (0.9)	0 (0)	8 (1.1)	1 (0.9)	1 (0.9)	1 (2.2)
**Sex, n (%)**								
Male	201 (39.1)	87 (30.4)	46 (43.4)	20 (42.6)	262 (36.8)	37 (33.0)	45 (42.1)	19 (42.2)
Female	306 (59.5)	194 (67.8)	54 (50.9)	26 (55.3)	438 (61.5)	74 (66.1)	56 (52.3)	25 (55.6)
Missing	7 (1.4)	5 (1.8)	6 (5.7)	1 (2.1)	12 (1.7)	1 (0.9)	6 (5.6)	1 (2.2)
**Diagnosis, n (%)**								
Schizophrenia and psychosis	238 (46.3)	85 (29.7)	23 (21.7)	11 (23.4)	297 (41.7)	34 (30.4)	22 (20.6)	10 (22.2)
Affective disorder	130 (25.3)	68 (23.8)	57 (53.8)	14 (29.8)	178 (25.0)	23 (20.5)	56 (52.3)	17 (37.8)
Other diagnosis[c]	138 (26.9)	128 (44.8)	24 (22.6)	21 (44.7)	225 (31.6)	54 (48.2)	26 (24.3)	17 (37.8)
Missing	8 (1.6)	5 (1.8)	2 (1.9)	1 (2.1)	12 (1.7)	1 (0.9)	3 (2.8)	1 (2.2)
**Relative in PPE, n (%)**								
Partner	161 (31.3)	100 (35.0)	42 (39.6)	13 (27.7)	233 (32.7)	32 (28.6)	37 (34.6)	16 (35.6)
Parent	269 (52.3)	138 (48.3)	33 (31.1)	21 (44.7)	364 (51.1)	58 (51.8)	36 (33.6)	17 (37.8)
Son/daughter	41 (8.0)	29 (10.1)	23 (21.7)	8 (17.0)	55 (7.7)	14 (12.5)	26 (24.3)	7 (15.6)
Sibling	18 (3.5)	9 (3.2)	2 (1.9)	2 (4.3)	24 (3.4)	5 (4.5)	2 (1.9)	2 (4.4)
Other relation	25 (4.9)	9 (3.2)	6 (5.7)	2 (4.3)	35 (4.9)	3 (2.7)	6 (5.6)	2 (4.4)
Missing	0 (0)	1 (0.4)	0 (0)	1 (2.1)	1 (0.1)	0 (0)	0 (0)	1 (2.2)
**Region, n (%)**								
Capital Region of Denmark	100 (19.5)	48 (16.8)	30 (28.3)	9 (19.2)	140 (19.7)	19 (17.0)	30 (28.0)	8 (17.8)
Central Denmark Region	113 (22.0)	64 (22.4)	32 (30.2)	13 (27.7)	151 (21.2)	29 (25.9)	32 (29.9)	11 (24.4)

North Denmark Region	57 (11.1)	37 (12.9)	14 (13.2)	4 (8.5)	83 (11.7)	15 (13.4)	14 (13.1)	6 (13.3)
Region Zealand	46 (9.0)	42 (14.7)	9 (8.5)	3 (6.4)	67 (9.4)	22 (19.6)	8 (7.5)	3 (6.7)
Region of Southern Denmark	198 (38.5)	95 (33.2)	21 (19.8)	18 (38.3)	271 (38.1)	27 (24.1)	23 (21.5)	17 (37.8)
Missing	0 (0)	0 (0)	0 (0)	0 (0)	0 (0)	0 (0)	0 (0)	0 (0)

[a] Patient-reported improvement, missing data: n=46 (5,4 %, outpatient), n= 9 (5,6 %, inpatient)

[b] Patient-reported satisfaction, missing data: n= 22 (2,6 %, outpatient), n=10 (6,1%, inpatient)

[c] Organic mental disorders, mental and behavioural disorders due to psychoactive substance use, neurotic, stress-related and somatoform disorders, behavioural syndromes associated with physiological disturbances and physical factors, disorders of adult personality and behavior, and other disorder/symptom (e.g. unclear diagnosis).

Additional table 1b. Association between caregiver involvement and patient-reported improvement: incl. region

	**Outpatient care: high patient-reported improvement**			**Inpatient care: high patient-reported improvement**		
Caregiver involvement	n (%)	Crude OR (95 % CI)	Adjusted OR (95 % CI)[a]	n (%)	Crude OR (95 % CI)	Adjusted OR (95 % CI)[a]
Staff supports patient in having contact with caregivers						
- High	360 (69.1)	2.10 (1.46-3.04)	2.22 (1.50-3.26)	65 (76.5)	2.34 (1.02-5.36)	2.37 (0.89-6.32)
- Low/none	84 (51.5)	Reference	Reference	25 (58.1)	Reference	Reference
Sufficient information about disease and treatment						
- High	233 (71.9)	1.88 (1.36-2.58)	1.75 (1.23-2.48)	36 (80.0)	2.25 (0.99-5.13)	2.31 (0.86-6.23)
- Low/none	236 (57.7)	Reference	Reference	64 (64.0)	Reference	Reference
Talk to staff about expectations						
- High	85 (71.4)	1.57 (1.00-2.47)	1.42 (0.86-2.33)	17 (68.0)	1.03 (0.40-2.67)	1.24 (0.42-3.68)
- Low/none	331 (61.4)	Reference	Reference	74 (67.3)	Reference	Reference
Staff ask about your experiences						
- High	187 (73.1)	1.97 (1.40-2.76)	1.78 (1.24-2.54)	31 (81.6)	2.50 (0.92-6.83)	2.96 (0.83-10.56)
- Low/none	274 (57.9)	Reference	Reference	69 (63.9)	Reference	Reference
Sufficiently involved in decision making						
- Yes	185 (70.1)	1.94 (1.33-2.82)	1.93 (1.28-2.90)	43 (81.1)	4.01 (1.69-9.51)	3.56 (1.33-9.57)
- No	140 (54.7)	Reference	Reference	30 (51.7)	Reference	Reference

[a] Adjusted for patient age, sex, diagnosis and region.

Additional table 1c. Association between caregiver involvement and patient-reported satisfaction with care: incl. region

	Outpatient care: high patient-reported satisfaction			Inpatient care: high patient-reported satisfaction		
**Caregiver involvement**	**n (%)**	**Crude OR (95 % CI)**	**Adjusted OR (95 % CI)[a]**	**n (%)**	**Crude OR (95 % CI)**	**Adjusted OR (95 % CI)[a]**
Staff supports patient in having contact with caregivers						
- High	490 (91.6)	3.75 (2.32-6.06)	4.23 (2.57-6.96)	64 (76.1)	2.53 (1.10-5.81)	3.44 (1.23-9.64)
- Low/none	125 (74.4)	Reference	Reference	24 (55.8)	Reference	Reference
Sufficient information about disease and treatment						
- High	303 (92.7)	2.85 (1.73-4.70)	2.77 (1.65-4.63)	38 (82.6)	2.76 (1.18-6.45)	2.80 (1.09-7.21)
- Low/none	350 (81.5)	Reference	Reference	62 (63.3)	Reference	Reference
Talk to staff about expectations						
- High	112 (90.3)	1.70 (0.86-3.38)	1.71 (0.83-3.54)	18 (69.2)	1.06 (0.41-2.73)	0.97 (0.35-2.65)
- Low/none	471 (84.6)	Reference	Reference	74 (67.9)	Reference	Reference
Staff ask about your experiences						
- High	240 (92.7)	2.59 (1.50-4.48)	2.44 (1.39-4.28)	33 (84.6)	3.20 (1.09-9.36)	3.91 (1.24-12.26)
- Low/none	409 (83.0)	Reference	Reference	67 (63.2)	Reference	Reference
Sufficiently involved in decision making						
- Yes	244 (89.1)	1.80 (1.06-3.06)	1.68 (0.95-2.97)	45 (81.8)	4.03 (1.70-9.56)	3.56 (1.37-9.23)
- No	212 (81.9)	Reference	Reference	29 (52.7)	Reference	Reference

[a] Adjusted for patient age, sex, diagnosis and region.
