# Supplementary material for: Family involvement and patient-experienced improvement and satisfaction with care: a nationwide cross-sectional study in Danish psychiatric hospitals
Source: BMC Psychiatry. 2021 Apr 13;21:190. doi: 10.1186/s12888-021-03179-1 (PMC8042926; doi:10.1186/s12888-021-03179-1)
Supplement: Supplementary file 4 — Additional file 4. Results of a sensitivity analysis according to non-appointed, non-responding and responding caregivers (outpatients). Results of a sensitivity analysis comparing descriptive statistics between patients with no appointed caregivers, appointed non-responding caregivers and appointed responding caregivers with regard to baseline characteristics and patient-reported improvement and overall satisfaction with care. This file only includes patients receiving outpatient care. [file 12888_2021_3179_MOESM4_ESM.pdf]

Additional file 4. Results of a sensitivity analysis according to non-appointed, non-responding and responding caregivers (outpatients)

Table.
Outpatient psychiatric care: descriptive characteristics for 6229 patients[a] according to caregiver participation

**Patient characteristics n=6229 (100 %)**	**No invited caregivers n = 4018 (64.5 %)**	**Responding caregivers n = 945 (15.2 %)**	**Non-responding caregivers n = 1266 (20.3 %)**
**Age, m (sd)**	40.3 (15.7)	38.9 (17.7)	37.7 (15.9)
Age missing, n (%)	160 (4)	13 (1)	28 (2)
**Sex, n (%)**			
Male	1422 (35)	335 (35)	407 (32)
Female	2455 (61)	596 (63)	838 (66)
Missing	141 (4)	14 (1)	21 (2)
**Diagnosis, n (%)**			
Schizophrenia and psychosis	1185 (29)	340 (36)	418 (33)
Affective disorder	866 (22)	247 (26)	334 (26)
Other diagnosis	1628 (41)	342 (36)	489 (39)
Missing	339 (8)	16 (2)	25 (2)
**Relationship, n (%)**			
Partner		342 (36)	518 (41)
Parent		429 (45)	461 (36)
Son/daughter		92 (10)	69 (5)
Sibling		32 (3)	50 (4)
Other relation		48 (5)	82 (6)
Missing		2 (0)	86 (7)
**Patient-reported improvement, n (%)**			
Low/none	1483 (37)	320 (34)	481 (38)
High	2132 (53)	564 (60)	679 (54)
Missing	403 (10)	61 (6)	106 (8)
**Patient-reported satisfaction, n (%)**			
Low/none	685 (17)	129 (14)	177 (14)
High	3152 (78)	789 (83)	1045 (83)
Missing	181 (5)	27 (3)	44 (3)

[a] Patients are only included with one observation disregarding that the patients may have two caregivers responding to the caregiver-questionnaire.
