# Supplementary material for: Family involvement and patient-experienced improvement and satisfaction with care: a nationwide cross-sectional study in Danish psychiatric hospitals
Source: BMC Psychiatry. 2021 Apr 13;21:190. doi: 10.1186/s12888-021-03179-1 (PMC8042926; doi:10.1186/s12888-021-03179-1)
Supplement: Supplementary file 5 — Additional file 5. Results of a sensitivity analysis according to non-appointed, non-responding and responding caregivers (inpatients). Results of a sensitivity analysis comparing descriptive statistics between patients with no appointed caregivers, appointed non-responding caregivers and appointed responding caregivers with regard to baseline characteristics and patient-reported improvement and overall satisfaction with care. This file only includes patients receiving inpatient care. [file 12888_2021_3179_MOESM5_ESM.pdf]

## Additional file 5. Results of a sensitivity analysis according to non-appointed, non-responding and responding caregivers (inpatients)

**Table. Inpatient psychiatric care: descriptive characteristics for 2010 patients[a] according to caregiver participation**

| **Patient characteristics n=2010 (100 %)** | **No invited caregivers n = 1596 (79.4 %)** | **Responding caregivers n =155 (7.7 %)** | **Non-responding caregivers n = 259 (12.9 %)** |
|---|---|---|---|
| **Age, m (sd)** | 42.4 (16.4) | 46.5 (19.4) | 42.1 (17.7) |
| Age missing, n (%) | 136 (9) | 2 (1) | 9 (3) |
| **Sex, n (%)** | | | |
| Male | 736 (46) | 66 (43) | 90 (35) |
| Female | 748 (47) | 84 (54) | 155 (60) |
| Missing | 112 (7) | 5 (3) | 14 (5) |
| **Diagnosis, n (%)** | | | |
| Schizophrenia and psychosis | 521 (33) | 35 (23) | 59 (23) |
| Affective disorder | 297 (19) | 67 (43) | 87 (34) |
| Other diagnosis | 535 (34) | 48 (31) | 98 (38) |
| Missing | 243 (15) | 5 (3) | 15 (6) |
| **Relationship, n (%)** | | | |
| Partner | | 58 (37) | 72 (28) |
| Parent | | 54 (35) | 87 (34) |
| Son/daughter | | 28 (18) | 35 (14) |
| Sibling | | 4 (3) | 25 (10) |
| Other relation | | 10 (6) | 18 (7) |
| Missing | | 1 (1) | 22 (8) |
| **Patient-reported improvement, n (%)** | | | |
| Low/none | 499 (31) | 49 (32) | 73 (28) |
| High | 915 (57) | 96 (62) | 161 (62) |
| Missing | 182 (11) | 10 (6) | 25 (10) |
| **Patient-reported satisfaction, n (%)** | | | |
| Low/none | 396 (25) | 44 (28) | 54 (21) |
| High | 1031 (65) | 101 (65) | 189 (73) |
| Missing | 169 (11) | 10 (6) | 16 (6) |

[a] Patients are only included with one observation disregarding that the patients may have two caregivers responding to the caregiver-questionnaire.
